# Induction of the pneumococcal *vncRS* operon by lactoferrin is essential for pneumonia

**DOI:** 10.1080/21505594.2018.1526529

**Published:** 2018-09-24

**Authors:** Seungyeop Lee, Prachetash Ghosh, Hyogyoung Kwon, Sang-Sang Park, Gyu-Lee Kim, Sang-Yoon Choi, Eun-Hye Kim, Thao Dang-Hien Tran, Seung Han Seon, Nhat Tu Le, Hamid Iqbal, Sangho Lee, Suhkneung Pyo, Dong-Kwon Rhee

**Affiliations:** a School of Pharmacy, Sungkyunkwan University, Suwon, Korea; b Soonchunhyang Institute of Medi-bio Science, Soonchunhyang University, Cheonan, Korea; c Department of Biological Sciences, Sungkyunkwan University, Suwon, Korea

**Keywords:** *Streptococcus pneumoniae*, VncRS, Pep27, lactoferrin, pneumococcal pneumonia

## Abstract

*Streptococcus pneumoniae* (pneumococcus), the major pathogen for pneumonia, commonly colonizes the lung, but the mechanism underlying the coordination of virulence factors during invasion via the host protein remains poorly understood. Bacterial lysis releases the components of the cell wall, and triggers innate immunity and the subsequent secretion of pro-inflammatory cytokines. Previously, the virulence of the *pep27* mutant was shown to be attenuated as a feasible candidate for vaccine development. However, the role of *pep27* gene, belonging to the vancomycin-resistance locus (*vncRS* operon), in virulence, is largely unknown. This study demonstrates that transferrin in the host serum reduces the survival of the host during *S. pneumoniae* infections in mice. The exposure of the pneumococcal D39 strain to lactoferrin induced the *vncRS* operon, lysis, and subsequent *in vivo* cytokine production, resulting in lung inflammation. However, these responses were significantly attenuated in pneumococci harboring a mutation in *pep27*. Mechanistically, the VncS ligand, identified as lactoferrin, induced the *vncRS* operon and increased the *in vivo* mortality rates. Thus, serum-induced activation of *vncRS* plays an essential role in inducing pneumonia.

## Introduction

Bacteria utilize numerous virulence factors for facilitating colonization. Components of bacterial cells comprise specialized secretion systems that resist the host immune system and sense the host environment, and respond by initiating a defined program that activates a virulence system [1]. Several two-component systems (TCSs) execute distinct functions that promote microbial survival. However, these proteins are also linked to virulence [2]. Bacteria can adapt to environmental changes using their TCSs [3]; however, the simple inactivation of the TCS may not substantially attenuate virulence owing to the redundancies in the regulatory systems. Additionally, which TCS plays a pivotal role in bacterial virulence is not completely understood.

Patients with iron deficiency anemia have higher levels of transferrin than normal individuals [4]. Lactoferrin (LF) is a multifunctional protein belonging to the transferrin family. The levels of transferrin have been shown to increase in iron deficiency anemia [5,6]. Additionally, incidents of bleeding in cancer patients can result in iron deficiency anemia, which subsequently increases the levels of lactoferrin [7–9]. Furthermore, patients with iron deficiency anemia are about four times more susceptible to contracting pneumonia than non-anemic individuals. However, the mechanism underlying this is yet to be experimentally determined [10].

Pneumonia is a highly prevalent acute respiratory disease with high morbidity and mortality rates that resulted in over 1.3 million deaths in 2010 alone [11]. The major causative agent of pneumonia, *Streptococcus pneumoniae* (the pneumococcus), asymptomatically colonizes the nasopharynx and causes potentially life-threatening infections, including sepsis and meningitis [12]. Over 50% of the cases of sepsis are secondary infections arising as a consequence of pneumonia [13]; however, the initiation of pneumococcal pneumonia and sepsis are poorly understood.

The role of VncRS in the pneumococcal virulence was still unidentified. The recent model suggested that Pep27, encoded upstream of the VncRS locus was secreted from the cell by the Vex123 transporter [14]. In this study, we elucidated the underlying immunological processes and identified VncRS, which is the vancomycin-resistant TCS [15] in serotype 2 of the D39 strain, to be responsible for fulminant inflammation and sepsis in mice, and as a key factor for lysis and sepsis. We additionally identified that mutations in *pep27*VncRS abrogate inflammation and sepsis.

## Results

### The vncRS operon of *S. pneumoniae* is critical for pneumococcal sepsis

**Figure 1.**
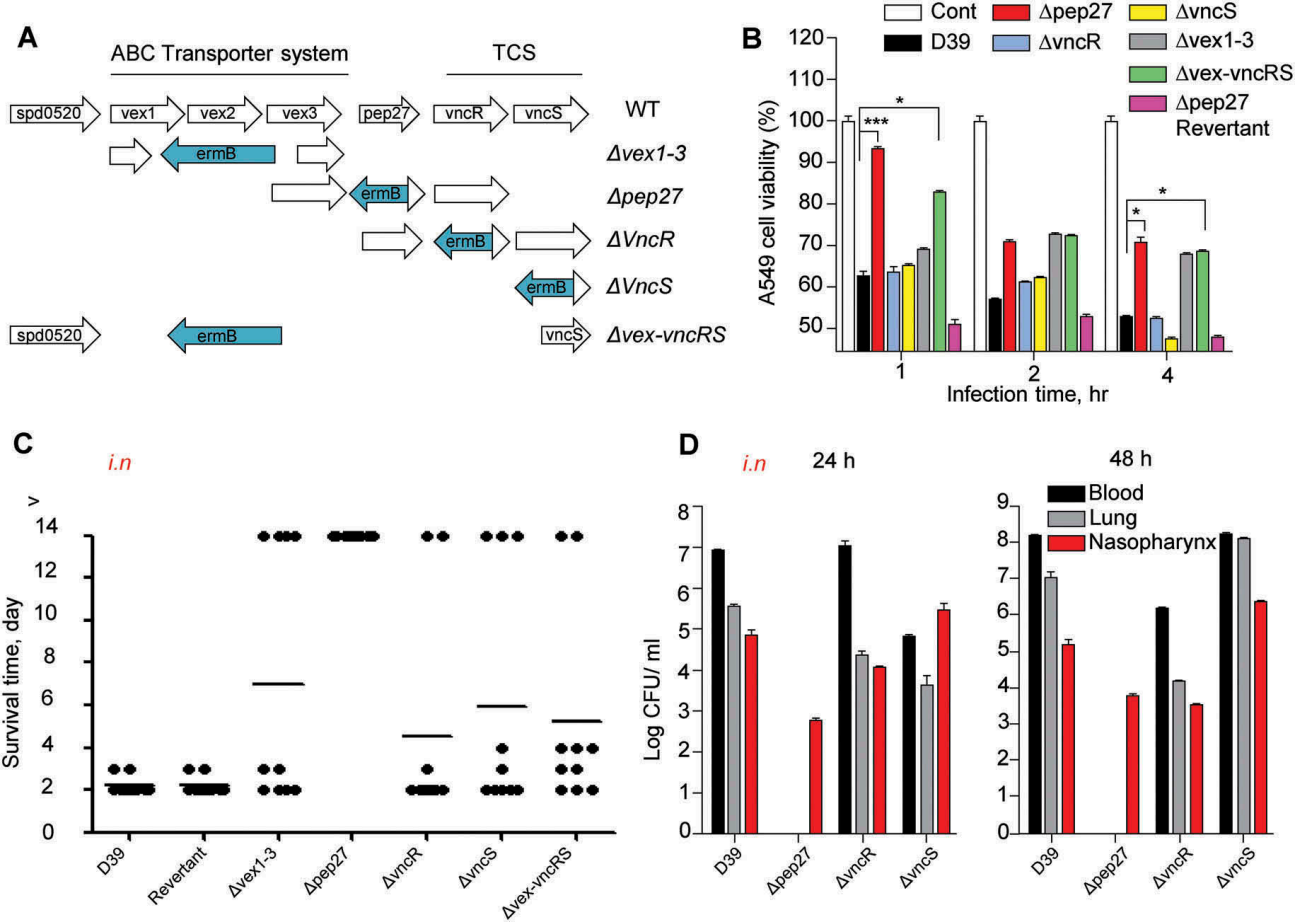
The necessity of Pep27 for virulence. (a) The target gene(s) were deleted and replaced by the erythromycin resistance cassette *ermB* in an orientation opposite to the direction of transcription by homologous recombination to prevent a polar effect. (b) A549 cells were infected with the mutants, and cytotoxicity was determined using the lactate dehydrogenase (LDH) assay. (c). Mice (CD1, *n *= 10 per group) were infected by the intranasal (*i.n*.) administration of 1–2 × 10^7^ CFU of the WT D39 or its isogenic mutants, and the survival rates were determined. (d) The WT D39 and various isogenic mutants were infected by *i.n*. administration, and the number of viable cells was determined 24 or 48 h post-infection. Except (a), all the data are expressed as the mean ± standard error of mean (SEM) of 3 experiments performed in duplicate. *P < 0.05 (one-way ANOVA).

Pep27 mediates LytA-dependent and LytA-independent lysis [14], and the *pep27* mutant (*Δpep27*) was not detected in the blood or brain after *i.n*. infections, showing significantly attenuated virulence [16]. This suggested that Pep27 might play a crucial role in inflammation and that pneumococcal lysis might be mediated by the serum [14]. To check whether the *vncRS* operon is critical for virulence, mutants carrying deletions in the *vncRS* operon were constructed (Figure 1(a)) and used for *in vitro*cytotoxicity and *in vivo* virulence assays. The *in vitro* infection of human A549 cells of the lung showed that the cytotoxicity of *Δpep27* cells was significantly attenuated and the attenuation was higher than both *ΔvncS* and *ΔvncR* cells (Figure 1(b)). To confirm the *in vivo*attenuation of cytotoxicity, mice (*n *= 10) were infected by the *i.n*. administration of 1–2 × 10^7^ CFU of WT D39 cells or the isogenic mutants, and the survival time was determined. Among the mutants, virulence was most significantly attenuated in *Δpep27*, in comparison to the WT (Figure 1(c)). To confirm the attenuation of virulence by *Δpep27*, a *Δpep27*revertant was constructed for use in further studies. The cytotoxicity exhibited by the *Δpep27* revertant was as high as the WT in both *in vitro* and *in vivo* experiments, in contrast to the *Δpep27* mutant (Figure 1(b, c)), which indicated that *pep27* is essential for achieving complete virulence. Since mutants with the deletion of the entire VncRS operon (*Δvex-vncRS*) still retained some virulence (Figure 1(c)), it is probable that virulence might be controlled not only by the VncRS operon but also by other redundant pathways. To corroborate the *in vivo* attenuation of virulence, the viable cells were counted to estimate the colonization of the mutants following *i.n*. infection. Following infection with *Δpep27*, no viable cells were detected in the lungs or blood 24 h post-infection, whereas a substantial number of viable cells were observed even after infection with the *ΔvncR* or *ΔvncS* (Figure 1(d)). None of the mice showed mortality following infection with *Δpep27* by *i.n*. or *i.v*. routes of administration, and we were unable to detect *Δpep27* at 24 h after *i.n*. infection [16]. This suggested that the *Δpep27* cells were unable to invade the lungs and blood from the nasopharynx and/or were rapidly cleared from the body.

### The vncRS operon is induced by serum

**Figure 2.**
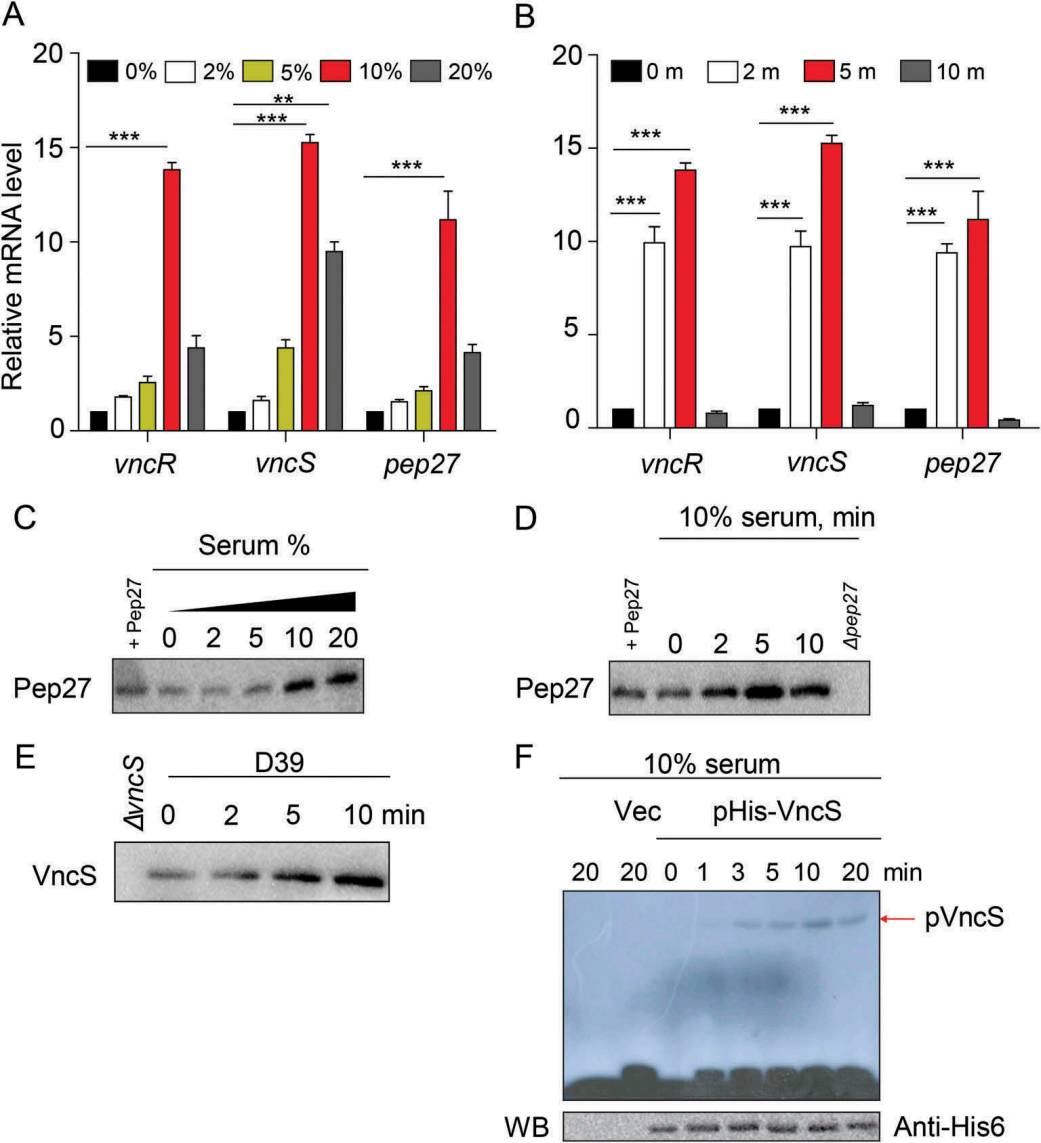
Serum-dependent inductionof *vncR/S* and autophosphorylation of VncS. (a–d) Wild type (WT) D39 cells were cultured in THY broth up to the mid-log phase (OD_550_ = 0.3), and then incubated with human serum for 5 min (a, c) or with 10% human serum for the durations specified (b, d). The mRNA levels were analyzed by qRT-PCR (a, d), and the Pep27 levels were determined by analyses with western blotting (c, d). (e) WT D39 cells at the mid-log phase were exposed to 10% human serum for the specified durations, and the protein levels were determined by analysis with western blotting. (f) The VncS expressed in *E. coli* (pHis-VncS) cells or the empty plasmid vector without any insert (Vec, control) was used for the auto-phosphorylation assays in the presence of [γ-^32^p] ATP. The loading dose of the VncS protein was measured by immunoblotting assays using anti-His_6_ antibody. (c, d, e, f) Representative data of at least 3 independent experiments. (a, b) The data are expressed as the mean ± standard error of the mean (SEM) of 3 experiments performed in quadruplicate. *P < 0.05 (one-way ANOVA).

To determine whether the expression of the *vncRS* operon is modulated by the serum during the invasion of pneumococci into the blood, the expression of the *vncRS* operon (*vex123-pep27-VncRS*) was examined in WT D39 cells. The D39 cells were cultured in THY broth until the mid-log phase (OD_550_ = 0.3) and supplemented with serum for 20 minor 10% human serum for the durations specified (Figure 2(a, b)). To confirm that these genes had been induced at the protein level, 10% serum was added to the mid-log phase of the cell culture, and analyzed with western blotting. Both Pep27 (Figure 2(c, d)) and VncS (Figure 2(e)), were induced by the supplementation of the serum, and Pep27 was secreted. Following host invasion, pneumococci are exposed to the host’s serum. The TCSs are autophosphorylated upon contact with the ligand [3]. Therefore, this study aimed to identify whether the autophosphorylation of TCSs occur after the VncS sensor kinase senses signals from the host serum. Our results showed that exposure to 10% serum results in autophosphorylation of the VncS in a time-dependent manner (Figure 2(f)), suggesting that the VncS sensor first perceives the signals from the host serum after which VncS is autophosphorylated.

### Pep27 contributes to inflammation during *S. pneumoniae* infection

**Figure 3.**
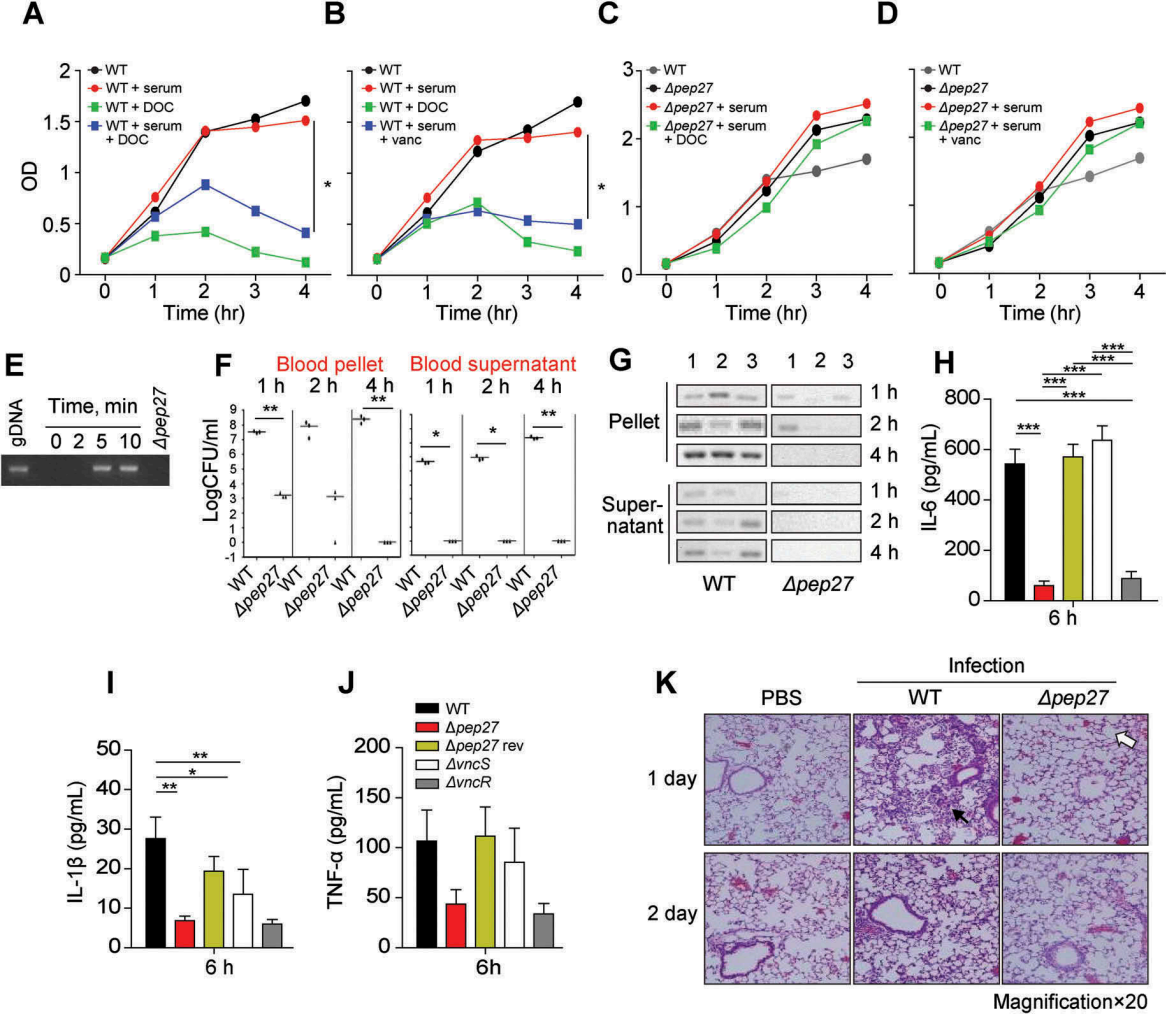
Pep27 is essential for lysis and secretion of proinflammatory cytokines. (a–d) Serum and/or lysis inducers were added to the cultures of WT D39 (a, b) or Δ*pep27* (c, d) grown in THY broth in the early exponential phase, and the OD was monitored. When required, the lysis inducers, deoxycholate (DOC, 100 µg ml^−1^) and vancomycin (0.4 µg ml^−1^), were added. (e) The WT D39 cells in the mid-log phase were exposed to 10% human serum for the specified durations, and the cell culture supernatant containing the released chromosomal DNA was detected by PCR using 16S rRNA primers. (f, g) CD1 mice (*n *= 3 per group) were infected by intravenous (*i.v.)* administration of either the WT D39 cells (7 × 10^7^ CFU) or mutant D39 *Δpep27* cells (1 × 10^8^ CFU). The blood samples were differentially centrifuged to separate the bacteria bound to the host cells (pellet) from the unbound bacteria collected in the supernatant, and the number of bacteria were enumerated by plating on blood agar (F). The chromosomal DNA released into the supernatant was detected by PCR using 16s rRNA primers (g). (h–j) Balb/c mice (*n *= 7 per group) were infected with 2 × 10^8^ CFU of the WT pneumococcal strain and the lung homogenates were used for determining the cytokine levels by ELISA. (k) CD1 mice were intratracheally infected with the WT D39 strain (1 × 10^7^ CFU) or the D39 mutant Δ*pep27* (2 × 10^8^ CFU). Lung sections at 1 or 2 days post-infection were stained with hematoxylin and eosin. All the data are expressed as the mean ± standard error of the mean (SEM) of a minimum 3 of independent experiments except (g) (a–d, error bars are smaller than the symbols used here). *P < 0.05 (a–d, f: one-way ANOVA, h–j: two-way ANOVA). (k) Representative data of at least 3 independent experiments.

In the presence of serum, supplementation with DOC drastically decreased the OD of the WT culture but not of the *Δpep27* culture (Figure 3(a–c)). Similarly, treatment with vancomycin decreased the OD (Figure 3(b–d)). These observations suggested that the *Δpep27* cells are lysis resistant. The major factors causing pneumococcal lysis are LytA and Pep27. However, in the vancomycin environment, the mRNA levels of *pep27* are maintained, while the levels of *lytA* are abruptly decreased [17]. In addition, in this study, the comparative analysis of LytA expression between WT and *Δpep27* strains revealed that the LytA levels were significantly higher in the WT strain (Suppl Figure 1). To corroborate pneumococcal lysis by the induction of VncRS, D39 cells cultured to the mid-log phase, were exposed to 10% serum and the chromosomal DNA released into the cell culture supernatant was detected by PCR using 16s rRNA primers. After adding sera, chromosomal DNA was detected in the cell culture supernatant (Figure 3(e)). To check lysis *in vivo*, WT D39 cells (7 × 10^7^ CFU) or mutant *Δpep27* cells (1 × 10^8^ CFU) were injected by *i.v*. administration into mice, and the number and lysis of bacterial cells was determined. The number of WT bacteria that were bound to the blood corpuscles and had been separated by centrifugation, substantially increased up to 4 h after injection, whereas the *Δpep27* cells bound to the blood corpuscles decreased to nil over the same period of time. Similarly, the WT cells in the supernatant that had been collected after centrifugation of human blood and comprised of unbound bacteria, showed a continuous increase in bacterial load over time, whereas the *Δpep27* cells did not show any bacterial load (Figure 3(f)). Additionally, in contrast to the continuous growth of the WT cells, the *Δpep27* cellswere rapidly cleared from the blood within 4 h after infection. Chromosomal DNA was detected in the supernatants after infection with WT bacteria 4 h post-injection, whereas negligible or no chromosomal DNA was detected in the supernatant after infection with *Δpep27*, suggesting that this mutant is not capable to induce lysis and hence no chromosomal DNA could be detected in supernatant (Figure 3(g)), indicating that the *Δpep27* cells were rapidly cleared. Since the proinflammatory cytokines produced within 6 h post-infection play a critical role in septic shock [18–21], the lysis-resistance of bacterial cells was corroborated by measuring the proinflammatory cytokines 6 h after *i.n*. infection. The cytokines were significantly induced in the lungs following infection with the WT cells, whereas the induction of cytokines was marginal or completely abolished following infection with *Δpep27*, possibly due to the rapid clearance of *Δpep27* cells (Figure 3(h–j)). These results indicated that the upregulation of cytokines at the very early stage of infection was probably due to lysis.

In order to evaluate the inflammation mediated by proinflammatory cytokines, the mobilization of eosinophils was analyzed using hematoxylin and eosin staining. Infection with the WT strain showed severe pulmonary inflammation with infiltration of larger inflammatory type cells (filled arrow in Figure 3(k)). In contrast, infection with *Δpep27* showed slight inflammation with neutrophil infiltration and the presence of open, regularly spaced alveoli (open arrow in Figure 3(k)).

### Pep27 mutations of *S. pneumoniae* impair sepsis in immunocompromised mice

**Figure 4.**
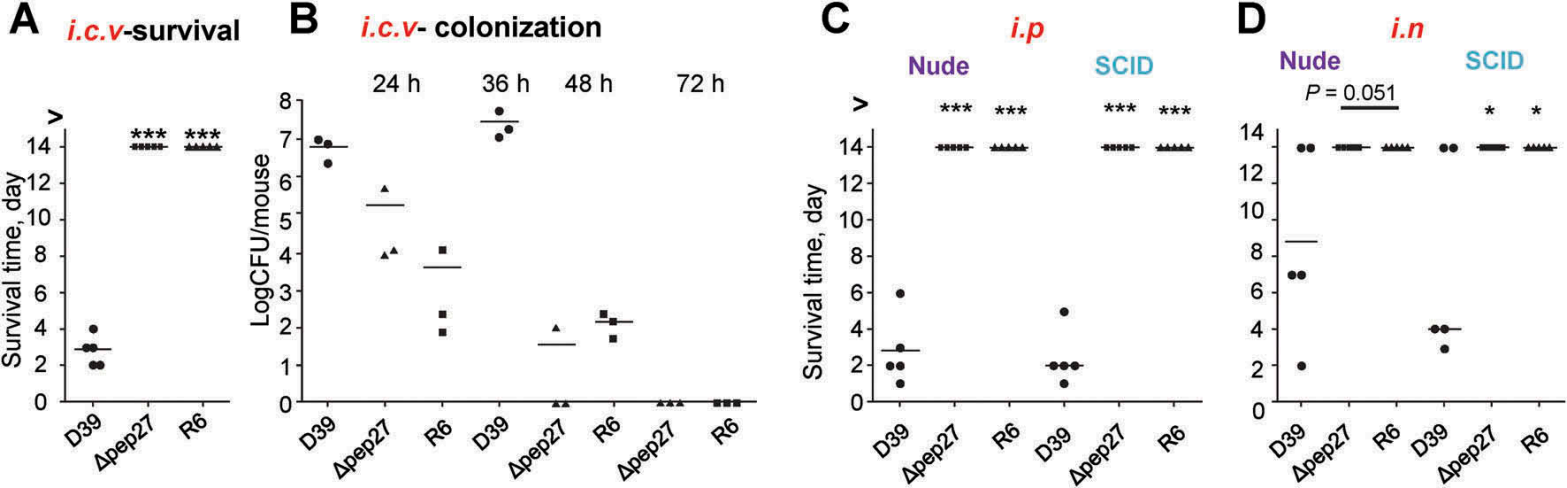
Infection with Δ*pep27* abolishes virulence as well as invasion into immunocompromised mice. (a) The attenuated virulence of *Δpep27* in the brain. CD1 mice (*n *= 5) were infected with the WT D39 (2 × 10^4^), mutant *Δpep27* (pep27 1 × 10^6^), or R6 (2 × 10^6^) strains via intracranial (*i.c.v*) administration, and the survival times were determined. (b) The rapid clearance of *Δpep27* observed in the brain. CD1 mice (*n *= 3) were infected with the WT D39 (5 × 10^3^), mutant *Δpep27* (1 × 10^4^), or R6 (7 × 10^3^) strains via *i.c.v*. administration, and the number of viable cells was determined. (c, d) Nude (T-cells deficient) or SCID (both B- and T-cell deficient) mice (*n *= 5) were infected by intraperitoneal (*i.p*.) administration of 1 × 10^4^ CFU (c), or intranasal (*i.n*.) administration of 1 × 10^7^ CFU of *Δpep27* (d), and survival times were determined. Each data point represents one mouse. The data are representative of 3 (a, b) or 2 (c, d) independent experiments. *P < 0.05 (one-way ANOVA) as compared between groups.

To corroborate the inability of *Δpep27* to invade other organs, the WT D39 cells (2 × 10^4^ CFU) and *Δpep27* mutants (1 × 10^6^ CFU) or non-virulent R6 cells (2 × 10^6^ CFU) were injected into the intracranial vein (*i.c.v*.), and the survival times were determined. Interestingly, although the number of viable *Δpep27*cells used for inducing infection was ~100-fold higher than the WT, infection with *Δpep27* did not result in mortality, whereas the mortality rate after injection of the WT cells was 100% (Figure 4(a)). Consistent with this observation, the *Δpep27* cells were cleared more rapidly than the WT cells, as observed from experiments on mice infected by the administration of the WT D39 cells (5 × 10^3^ CFU), *Δpep27* (1 × 10^4^ CFU), or R6 (7 × 10^3^ CFU) into the *i.c.v*. (Figure 4(b)). To corroborate the abrogation of virulence in the *Δpep27* mutant, nude mice (deficient in T-cells) and SCID mice (deficient in both B- and T-cells)were infected by *i.n*. administration of1 × 10^7^ CFU or *i.p*. administration (sepsis model) of 1 × 10^4^ CFU of *Δpep27*, respectively. Infection with *Δpep27* did not induce mortality in any of the immunocompromised mice, whereas infection with the WT induced mortality in all of the mice in the sepsis model and more than half of the mice that were infected by *i.n*. routes (Figure 4(c, d)), indicating the necessity of Pep27 for inducing inflammation and sepsis in the lung.

### vncRS and pep27 of *S. pneumoniae* are upregulated by transferrin in the host serum

**Figure 5.**
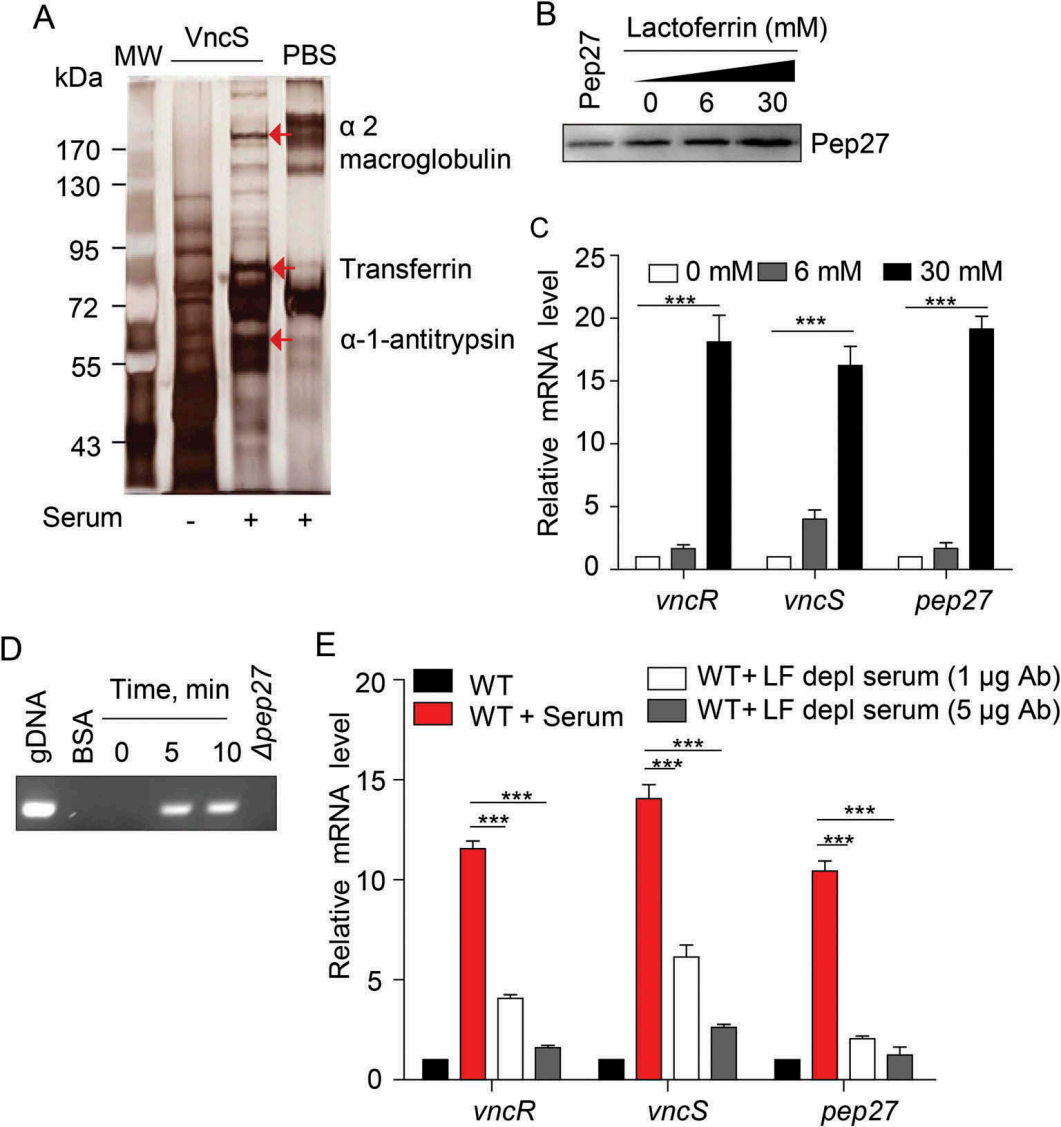
The VncS ligand, lactoferrin (LF), induces *vncR/S*, Pep27, and lysis. (a) Purified His_6_-VncS protein bound to a 3-dimensional Sol-gel® bed was incubated with human serum for screening VncS ligands. The bound ligand was subsequently subjected to SDS-PAGE and mass analyses. (b–d) The WT D39 strain was incubated at the mid-log phase with various concentrations of LF for 5 min (b, c) or with 30 mM LF for the specified duration (d). Western blotting (b) and qRT-PCR (c) were performed. Quantification of the released chromosomal DNA using the genomic DNA (gDNA) as a control (d). (e) The WT D39 strain was incubated for 5 min with LF in the LF-depleted human sera, which was prepared by adding either 1 or 5 μg of LF antibody to human serum. The mRNA levels were determined by qRT-PCR. The data in (a), (b), and (d) are representative of at least 3 independent experiments. The data in (C) and (e) are expressed as the mean ± standard error of the mean (SEM) of 3 experiments performed in quadruplicate. *P < 0.05 (one-way ANOVA).

The induction of *vncRS* by the addition of serum indicated that the VncS can sense the components of the serum and modulate virulence. In order to screen the VncS ligand, VncS was adsorbed onto a Sol-gel® bed and then incubated with human serum in a 3-dimensional array. The VncS ligand was subsequently dissociated from the Sol-gel® bed and subjected toSDS-PAGE. Mass analyses revealed that macroglobulin, transferrin, and antitrypsin in the serum act as VncS-binding proteins. However, these putative ligands alone did not bind to the Sol-gel® bed (Figure 5(a)). After adding these putative ligands to the culture broth, the induction of Pep27 was determined. Among the proteins, only LF, a member of the transferrin proteins in the host serum, induced Pep27 (Figure 5(b)), suggesting that LF might be a ligand of VncS. As we initially performed the western blot for VncS protein expression after alpha-1-antitrypsin and alpha 2 macroglobulin supplementation, and we could not detect any significant changes in VncS expression (Suppl fig. 2). Thus, we did not check the Pep27 induction.

To confirm whether LF induced the *vncRS* operon and triggered subsequent lysis, LF was added to the culture, and the induction of *vncRS* was determined by qRT-PCR. As expected, LF induced the *vncRS* operon (Figure 5(c)). Moreover, the supplementation of LF to the culture of WT strain induced lysis and released the chromosomal DNA into the supernatant, whereas, we could not detect any chromosomal DNA from the supernatant of *Δpep27* culture as deletion of this *pep27* could inhibit lysis (Figure 5(d)). Additionally, the supplementation of LF to the *Δpep27* revertant culture could also induce lysis and chromosomal DNA could also be detected in supernatant, suggesting that Pep27 was directly involved in this LF-mediated lysis (Suppl fig. 3). In addition, we could not detect any chromosomal DNA in the supernatant of LF-supplemented culture of other deletion mutants of VncRS operon (Suppl Figure 3), which was probably due to the lesser secretion of Pep27 from these mutants, which might be not sufficient to induce lysis. To confirm whether LF was indeed a VncS ligand, LF-depleted serum was used to determine any changes in the expression of *vncRS*. Analyses with qRT-PCR revealed that the depletion of LF abolished the induction of *vncRS* (Figure 5(e)).

### Lactoferrin deficient mice show high survival rate

**Figure 6.**
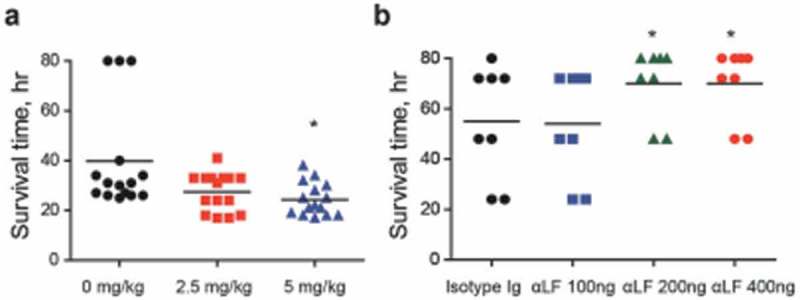
Lactoferrin (LF)-dependent sepsis. (a) LF was orally administered to mice (*n *= 15 per group), 6 h prior to lethal intraperitoneal (*i.p*.) infection with the WT D39 strain (2 × 10^4^ CFU), and survival rates were recorded every hour. (b) LF antibody was administered to mice (n = 8 per group) by *i.p*. administration at doses of 100, 200, and 400 ng/mouse and by *i.p*. route usingone-fourth of the dose used during intranasal (*i.n*.) administration at 25, 50, and 100 ng/mouse 24 h prior to the *i.n*. administration of the WT D39 strain (2 × 10^8^ CFU), and the survival rates were monitored. The data are expressed as the mean ± standard error of the mean (SEM) of 3 experiments performed in duplicate. *P < 0.05 (Log-rank test).

To verify LF as an *in vivo* VncS ligand, LF was orally administered to mice (n = 15 per group) 6 h prior to *i.p*. infection with WT D39 cells, and the survival rates were determined. The administration of LF significantly increased the mortality rate (Figure 6(a)). Furthermore, to evaluate the effect of the neutralizing antibody against *S .pneumoniae*, the depletion of LF prior to the *i.n*. infection (n = 8 per group) of the WT D39 strain (pneumonia model) decreased the mortality rate in a dose-dependent manner (Figure 6(b)), thus confirming that LF is a ligand of VncS and is necessary for survival during lung inflammation.

## Discussion

In a recent study, 17% for sepsis and 26% for severe sepsis were found to be responsible for the deaths at the hospital [22]. After abdominal and thoracic surgery, postoperative pulmonary complications are usually accompanied by pneumonia, pulmonary edema, and acute respiratory failure [23]. In addition, the association of anemia with greater extent of postoperative morbidity and mortality is also reported [24]. Our body tries to regulate transferrin levels to reach normalized hemoglobin in order to restore this anemia [25].These findings support the clinical importance of transferrin in iron-deficient anemia. As the anemic individuals are more prone to pneumococcal diseases, the serum transferrin must have a role to determine the host survival against pneumococcal infection.

Pneumococcal disintegration releases pneumolysin, cell wall components, and transforming DNA [26], which trigger the host immune system resulting in the production of proinflammatory cytokines, and subsequent inflammation and death [27].Thus, understanding the mechanism by which pneumococcal lysis is regulated is crucial for the development of novel chemotherapeutic strategies. Since pneumococcal lysis by antibiotics could result in the exacerbation of clinical symptoms, the treatment of pneumococcal pneumonia should be undertaken in such a way so as to avoid bacterial lysis [28]. However, the indicator(s) of pneumococcal lysis during infection and sepsis, and the process by which lysis is modulated remains unknown.

Studies on Pep27 have primarily focused on antibiotic resistance and antibiotic-dependent lysis [14,29,30]. Under the selective pressure of antibiotics, vancomycin-tolerant strains of *S. pneumoniae* exhibit tolerance to high concentrations of antibiotics by repressing the expression of *pep27* in the stationary phase of the growth curve, which ensures subsequent survival in the host resulting in therapeutic failure [17]. During transformation, a small subpopulation of pneumococci are initially lysed [26] and the pneumococcal DNA is detected after infecting human lung specimens *in vitro*, which is accompanied by the increased secretion of inflammatory cytokines [31]. Additionally, the inhibition of autolysis can prevent phagocytosis and cytokine production [32]. However, Pep27 itself does not show any cytotoxicity to human erythrocytes [33].Therefore, we suggested that the secretion of cytokines and inflammatory response during pneumococcal infections appear to depend on lysis, and that the induction of lysis by *pep27*probably triggers the production of cytokines and inflammation, resulting in pneumococcal invasion into the blood, leading to sepsis.

This study also demonstrated that neither the VncS sensor kinase nor the VncR response regulator significantly contributes to sepsis. However, the effector molecule Pep27 does contribute to sepsis, suggesting that Pep27 is a key contributor to inflammation and sepsis during pneumococcal infections. Moreover, VncS senses LF in the blood and induces the *vncRS* operon and subsequent lysis. The initial incoming signal, LF, the presence of which in the blood is strongly correlated to the occurrence of sepsis [34–36], was found to play a crucial role in lysis, albeit only a small percentage (<1%) of the bacterial population was lysed (Suppl Figure 4).

Thus, the lysis of a small population of bacterial cells in the lung microenvironment appears to be sufficient for initiating an inflammatory response and for mobilizing leukocytes and pathogens, resulting in the dissemination of pathogens, leading to sepsis and mortality.

Pneumococci are subject to phase variations after invading the blood from the nasopharynx [37]. Pneumococci are predominantly present in the nasopharynx, exhibiting a transparent phenotype with a thin capsule; however, the cells turn opaque after acquiring a thick capsule that protects from phagocytes, with the polysaccharide capsule being maximally synthesized in most of the pneumococci [37]. Our study suggests that, when pneumococci are exposed to the host serum by transcytosis or by invasion into the blood, a VncS sensor perceives LF, a component of the serum, resulting in the phosphorylation of VncS. Phosphorylated VncS subsequently delivers a phosphate group to VncR, and phosphorylated VncR binds to the promoters of target genes to induce or repress their expression, which allows the pneumococci to adapt to the milieu of the blood. Thus, it is highly probable that VncR-induced genes might encode components necessary for capsule synthesis. Further studies need to be performed for elucidating the underlying mechanism by which VncS senses LF and the mode of regulation of the target genes by VncR.

The results of this study will aid the development of chemotherapeutic agents for the prevention and treatment of pneumococcal sepsis and could exemplify the success of strategies intervening with either the VncS sensor or the VncR-dependent gene regulation. Since chemical intervention could agonize or antagonize the incoming signal and perturb or accelerate the process of lysis, it could be used as a platform for the development of chemotherapeutic agents and/or preventive strategies. *In silico* analyses would allow the design of target molecules that could antagonize either the VncS or VncR, by blocking the VncS signal and the downstream events of the signal transduction cascade, resulting in abrogation or reduction of pneumonia and/or sepsis.

## Methods

### Bacterial strains

The encapsulated *S. pneumoniae* serotype 2 of the D39 strain (NCTC7466) was donated by David Briles of the University of Alabama at Birmingham, USA. The *Δpep27* mutant of the D39 strain and the non-encapsulated CP1200 strain weregrown in Todd Hewitt broth or Casitone-tryptone (CAT)-based medium as previously described [16]. The transformation medium contained CAT broth, 147 mg of CaCl_2_, and 2 g of bovine serum albumin (fraction V; Sigma) per liter. Competence stimulating peptide-1 was added in order to induce competence, as previously described [38].

### *In vivo* infection studies

For *in vivo* survival and colonization studies, 4-week-old male CD1 and nude mice (Orient Bio Inc., Korea), or SCID mice (Charles River Laboratories, Japan) were used. The virulence of the *S. pneumoniae* strain was evaluated by intraperitoneal (*i.p*., 100 μl per mice), intranasal (*i.n*., 20 μl per mice), or intracranial (*i.c.v*., 5 μl per mice) challenge with a highly virulent type 2 D39 strain or its isogenic mutants,as previously described [16]. The use of animals in this study was approved by the Animal Ethical Committee of Sungkyunkwan University, in accordance with the guidelines of the Korean Animal Protection Law.

### Screening vncS ligands by sol-gel® analysis

To identify the VncS ligand(s) in human serum, purified VncS was conjugated to the Sol-gel® bed and incubated with human serum for 24 h; the entrapped ligand was retrieved by the gel (Sol-gel® method, PCL Inc., Korea) [39]. SDS-PAGE and mass-mass analyses were subsequently performed for identifying the VncS-binding proteins. This analysis was performed by PCL Inc., Korea.

### Cell cultures

The human lung epithelial carcinoma A549 cell line (American Type Culture CollectionATCC TIB-71) was cultured at 37°C in the presence of 5% CO_2_ and 95% air in Dulbecco modified Eagle’s medium (DMEM) supplemented with 10% fetal bovine serum (FBS) (Gibco BRL), 4.5 g l^−1^ glucose, 100 U ml^−1^ penicillin G, and 100 µg ml^−1^streptomycinas the basic medium.

### Construction of deletion mutants

In order to construct deletion mutants of the *vncRS* locus of *S. pneumoniae*, a donor DNA fragment containing a deletion in the locus of interest was replaced with an erythromycin resistance cassette (*ermB*) in a direction opposite to that of transcription by overlapping PCR, and was integrated into the pneumococcal chromosomes by homologous recombination [16]. The following mutants of the D39 strain were constructed by tripartite PCR and transformed: *ΔvncR* (left arm primers: 5ʹ-TATTGAGGCAGCGGACGGTCAG-3ʹ and 5ʹ-**ATCAAACAAATTTTGGGCCCGG**-CCACCTTGGTATCCTTGT-3ʹ; right arm primers: 5ʹ-**ATTCTATGAGTCGCTGCCGACT**-GACTCGGTCTCAGATTATCG-3ʹ, and 5ʹ-ATTGCGCACAGTGAGGATAC-3ʹ; deleted from nucleotides 535,257 ~ 535,359 of the D39 genome),*ΔvncS*(left arm primers: 5ʹ-AGGCAGAGTATCGAGCAAGT-3ʹ and 5ʹ-**ATCAAACAAATTTTGGGCCCGG**-CAATAGTCCGAGCGTAGATG-3ʹ; right arm primers: 5ʹ-**ATTCTATGAGTCGCTGCCGACT**-CTGTGCAGGAATTGCGAGAT-3ʹ and 5ʹ-GAACTAAGCCACCTGGAACAGA-3ʹ; deleted from nucleotides 536,012 ~ 536,389 of the D39 genome), *Δvex1*-3 (left arm primers: 5ʹ-GTCAGCAGAAAGCGACTGAG-3ʹ and 5ʹ-**ATCAAACAAATTTTGGGCCCGGG**-AGCCGTTAAGCTAACCAAG-3ʹ; right arm primers: 5ʹ-**ATTCTATGAGTCGCTGCCGACT**-CGGCCTCAAGCAGGCAAGTA-3ʹ and CGCCATAACGAGAACCACTA-3ʹ; deleted from nucleotides 531,534–534,177 of the D39 genome), and *Δvex1-vncS* (left arm primers: 5ʹ-GTCAGCAGAAAGCGACTGAG-3ʹ and 5ʹ-**ATCAAACAAATTTTGGGCCCGGG**-AGCCGTTAAGCTAACCAAG-3ʹ;right arm primers:5ʹ-**ATTCTATGAGTCGCTGCCGACT**-CTGTGCAGGAATTGCGAGAT-3ʹ and 5ʹ-GAACTAAGCCACCTGGAACAGA-3ʹ; deleted from nucleotides 531,534–536,389 of the D39 genome) [16]. The sequences represented in bold are complementary to *ermB*. The transformants were selected by adding 2.5 µg ml^−1^ erythromycin to the growth medium and were screened for the correct deletion using PCR, sequencing, and immunoblot analysis.

### Protein purification

The complete nucleotide sequence of VncS, encoding residues 1–442, was amplified by PCR using the primers VncS F (5ʹ-GGGCCC***GGATC*C**GATGAAACGAACAGGTTTATTTGC-3ʹ, the *Bam*HI site is italicized) and VncS R (5ʹ-GGCCCG***CTCGAG***CTAGTCTTGGACGACTTTTGG-3ʹ, the *Xho*I site is italicized) with the genomic DNA of *S. pneumoniae* D39 strain as template. The kinase domain of *vncS* (residues 194–442) was amplified by PCR using the VncS 194F (5ʹ-CGC***GGATCC***AAGGATGAGATAGGTAATCTCAAG-3ʹ, the *Bam*HI site is italicized) and VncS R primers. The amplified DNA was cloned into the *Bam*HI/*Xho*I-sites of the pHis Parallel 2 vector and transfected into *Escherichia coli* XL1-blue cells and subsequently transferred to the *E. coli* BL21 expression strain (DE3).

In order to purify the recombinant VncSprotein, the recombinant strains were grown at 37 ºC to an optical density (OD)_600_ of 0.6 to 0.8 in Luria Bertani (LB) with 50 μg ml^−1^ ampicillin. Protein expression was induced by the addition of isopropyl β-D-1-thiogalactopyranoside (IPTG) to the culture at a final concentration of 0.5 mM.However, upon addition of IPTG, the membrane proteins aggregated and their activities decreased [40]. Therefore, the entire VncS protein, comprising of residues 1–442, was cultured in the absence of IPTG. The cells were incubated at a steady temperature of 25 ºC for 24 h, collected by centrifugation at 3,382 × *g* for 10 min, and suspended in default buffer (50 mM Tris-HCl at pH 7.5, 150 mM NaCl, 5 mM β-mercaptoethanol, and 1 mM phenylmethylsulfonyl fluoride [PMSF]) with the complete VncS protein (residues 1–442) or with the kinase domain of VncS (residues 194–442) with 0.5% Triton X-100. The cells weresubsequently lysed by sonication on ice, and the cellular debris and insoluble proteins were removed by centrifugation at 16,000 × *g* for 1 h. The protein fractions in the supernatant were purified using a Ni-NTA resin (Qiagen) as previously described [41]. When necessary, the His_6_ tag was removed using 100 units of the tobacco etch virus protease during dialysis against 1 liter of default buffer along with 1 mM EDTA and 1 mM dithiothreitol overnight at 4 ºC. The His_6_ fragments and uncut proteins were removed using a Ni-NTA column. The protein was further purified using a mono-Q ion exchange column (50 mM Tris buffer, with or without 0.5% Triton X-100, and 1 M NaCl gradient) and a Superdex-200 size exclusion column (GE HealthCare) equilibrated with buffer B (50 mM Tris-HCl at pH 7.5, 150 mM NaCl, 1 mM DTT). Buffer C (50 mM Tris-HCl at pH 7.5, 150 mM NaCl, 1 mM DTT, and 1 mM EDTA, 0.5% Triton X-100) was used for purification. The purity of the purified protein was verified by SDS-PAGE and further concentrated to 10 mg ml^−1^ using a centrifugal filter (Centricon®, Millipore).

The complete sequence of VncR was cloned into the pHis Parallel vector and expressed similarly (details are available on request).

### Preparation of *E. coli* membranes with vncS

The *in vitro* ligand response of VncS was assayed using VncS-induced *E. coli* membranes that were purified as previously described, with minor modifications [42]. Briefly, VncS-induced *E. coli* cells were resuspended in membrane buffer (50 mM Tris-HCl at pH 7.5, 5 mM EDTA, 2 mM PMSF, and 10% v/v glycerol). The cells were lysed by sonication on ice. Following centrifugation at 20,000 × g for 10 min, the membranes were isolated from the supernatant by ultra-centrifugation at 100,000 × *g* for 60 min. The membrane pellet was suspended in membrane buffer and used for the *in vitro* auto-phosphorylation assay.

### Autophosphorylation of vncS

The kinase domain of VncS was assayed for phosphorylation activity, according to a previously reported method, with modifications [43]. The kinase activity was measured by phosphorylation assays performed at room temperature for 30 min, with a final sample volume of 25 μl that contained 0.25 μM of [γ-^32^P] ATP (specific activity, 3,000 Ci/mmol), 24.75 μM of ATP, 10 μL of phosphorylation buffer (50 mM Tris-HCl at pH 7.8 and 200 mM KCl), and 1 μg of VncS (amino acids 194–442). The *in vitro*VncS phosphorylation assay was carried out using 2 μg of VncS (residues 1–442) embedded in *E. coli* membranes with or without 10% human serum. The reaction was terminated by the addition of 5X SDS sample buffer, and subjected to SDS-PAGE using 15% polyacrylamide gels. The labeled proteins were detected using a Phosphor Imager film (Agfa, Belgium) using a 24 h exposure.

### RNA isolation and quantitative reverse transcription (qRT)-PCR

To determine gene expression at the mRNA level, pneumococci in the logarithmic phase of growth (OD_550_ = 0.30) were exposed to the serum or human LF (6 mM and 30 mM) for the specified time periods, and the total RNA was isolated using the Trizol method (Invitrogen, USA). The isolated RNA (1–2 µg) was used as a template for preparing cDNA, using the manufacturer’s instructions (Enzymonics, Korea). The qRT-PCR reaction was performed with a final sample volume of 20 µl, comprising of 50 ng of cDNA, 10 pmol of each primer, and the SYBR Green master mix (Elpis, Korea), using the StepOne Plus Real-Time PCR System (Applied Biosystems, USA). Primers 5ʹ-CCCCTTATGACCTGGGCTACA-3ʹ and 5ʹ-CGGCTTGCGACTCGTTGT-3ʹ were used for amplifying the 16s RNA, primers 5ʹ-ATGAGAAAGGAATTTCACAACG-3ʹ and 5ʹ-TCACGGATCATCTCTT-3ʹ were used to amplify *pep27*, primers 5ʹ-GGTGGCCCTGGTTTTACTGG-3ʹ and 5ʹ-ATCGCGTCCACCCTCACTTT-3ʹ were used for amplifying *vncR*, andprimers 5ʹ-CCAGCTGGAGAAGATGAAGG-3ʹ and 5ʹ-GTTCATCCACAATCCCCAAG-3ʹ were used to amplify*vncS*. During PCR, the temperature was initially maintained at 95°C for 10 min, followed by 40 cycles of 95°C for 15 s, 55°C for 30 s, and 72°C for 30 s. All real-time PCR data were normalized to the mRNA levels of the 16s rRNA gene, used as an internal control. The quantity of transcripts was expressed as the n-fold change compared to the internal control gene (2^−ΔCT^, where ΔCT stands for the alteration in threshold cycle between the target and internal control genes).

### *In vitro* cytotoxicity assay

The cytotoxicity of the mutant D39 strains on human A549 carcinoma cells was determined by measuring the lactate dehydrogenase (LDH) released from the damaged cells into the supernatant, by using the LDH Cytotoxicity Detection Kit (Takara Bio Inc., Tokyo, Japan), according to the manufacturer’s instructions.

### Immunization of mice

Mice were immunized according to the protocol described in a previous study [44], with slight modifications. Briefly, separate formulations of the His_6_-tagged VncS antigen and the commercially available synthetic Pep27 peptide (ProSci Inc, USA) were prepared using aluminum hydroxide (Alum) as an adjuvant, in the ratio 1:10. Balb/c mice were immunized three times at 2-week intervals, by the *i.p*. administration of10 μg of the individual formulations. One week after the final immunization, cardiac puncture was performed, and the polyclonal mouse sera were collected and stored at 4°C until use.

### Western blotting

The bacterial pellets and supernatants were subjected to analysis with SDS-PAGE. The separated proteins were transferred by electroblotting onto PVDF membranes, which were incubated with polyclonal mouse anti-Pep27 antibody (1:1000), anti-VncS antibody (1:3000), or anti-VncR antibody (1: 3000) that served as probes. The membranes were then incubated with goat anti-mouse IgG horseradish peroxidase-conjugated secondary antibody (Santa Cruz Biotechnology, USA) at a dilution of 1:4000. The labeled bands were visualized using an ECL reagent (Gendepot, USA) according to the manufacturer’s protocol.

### Detection of pep27 secretion and lysis

The wild-type (WT) *S. pneumoniae* D39 strain and its isogenic mutantswere cultured in THY broth until the cells reached the mid-log phase (OD_550_ = 0.3) and then grown in 1% FBS for 1 h. Also, the WT D39 strain was cultured in THY broth until the indicated OD value was reached. The culture was centrifuged, and the supernatant was filtered through a 0.22 nm filter and an ultra-Centricon (MWCO 10 kDa), followed by precipitation with trichloroacetic acid (TCA). Using the anti-Pep27 antibody, 20 μg of the proteins precipitated by TCA were analyzed by western blotting.

To detect bacterial lysis, the DNA from the bacterial chromosomes that had been released into the supernatant, was harvested by centrifugation at 21,000 × *g* for 10 min, which precipitated all of the intact bacterial cells into a pellet. The DNA from the bacterial chromosomes was detected by RT-PCR using 16s rRNA primers (16S-F: 5ʹ-CTGTGGCTTAACCATAGTAG-3ʹ, and 16S-R: 5ʹ-CTAGCACTCATCGTTTACA-3ʹ).

For the *in vivo* measurement of bacterial lysis, CD1 mice (*n *= 3 per group) were infected by the intravenous (*i.v*.) administration of either the WT D39 (7.2 × 10^7^ CFU) or *Δpep27* (3.7 × 10^7^ CFU). The blood samples weredifferentiallycentrifuged (1,500 × *g* for 10 min) to separate the bacteria bound to host cells (as pellets) from the unbound bacteria in the supernatant. The samples were subsequently appropriately diluted and plated onto blood agar for counting the number of viable cells.

### Autolysis in the presence of deoxycholate (DOC) and antibiotics

To determine the effect of the serum on cell lysis induced by antibiotics or the detergent DOC, the bacterial strains were allowed to grow in THY broth until the early exponential phase (at an OD_550_ of 0.15–0.17), at which time sodium deoxycholate (100 µg ml^−1^), vancomycin (0.4 µg ml^−1^), and 10% serum were added, and the OD, indicating the number of cells in each culture, was determined.

### Estimation of *in vivo* cytokine secretion

To evaluate the *in vivo* cytokine secretion, 6- to 8-week old male Balb/c mice were infected with 2 × 10^8^ CFU of WT *S. pneumoniae* D39 cells or its isogenic deletion mutants, and the lung homogenates were collected after 6 h and 24 h of infection. The quantity of IL-6, IL-1β, and TNF-α secreted in the pulmonary tissues was measured with commercially available enzyme-linked immunosorbent assay (ELISA) kits (eBioscience, USA), according to the manufacturer’s protocol.

### *In vitro*depletion of LF

To prepare LF-depleted human sera, 100 µl of human serum (Sigma-Aldrich, USA) was incubated overnight with 1 µg or 5 µg of polyclonal anti-LF antibody (Abcam, UK) at 4°C under gentle agitation. The immune complex was removed by pull down with 100 µl of protein A/G-PLUS agarose beads (Santa Cruz Biotechnology, USA). The supernatant served as the LF-depleted human serum.

### Determining the effect of LF on virulence *in vivo*

For evaluating the effect of LF on virulence, LF was orally administered to 6- to 8-week old male Balb/c mice 6 h prior to infection by the *i.p*. administration of 2 × 10^4^ CFU of WT *S. pneumoniae* D39 cells, and the rate of survival was monitored.

For the *in vivo* experiments on LF depletion,Balb/c mice were treated with *i.p*. (0, 100, 200, and 400 ng kg^−1^) and *i.n*. (0, 25, 50, and 100 ng kg^−1^) administration of LF-neutralizing IgG antibody (anti-mLactoferrin, Aviva Systems Biology, USA) or mouse IgG isotype control, 1 day prior to the infection with 2 × 10^8^ CFU of D39 cells, intranasally, and the survival rate was monitored.

### Statistical analyses

Statistical differences between the medians of the groups were analyzed by the two-tailed, unpaired, Mann-Whitney U test, and differences in the overall survival rates between the groups were analyzed by the Fisher’s Exact test.One-way or two-way analysis of variance (ANOVA) was also used for statistical analyses. The data are represented as the mean ± standard error of the mean (SEM) for 2–4 independent experiments. The data obtained from ELISA were expressed as the average derived from triplicatewells ± SEM. Statistically significant differences weredefined by *P *< 0.05.

## Data Availability

The authors declare that all the relevant data pertaining to the experiments described herein are presented within the article and the supplementary data files, and relevant data are available from the authors on request.
