# Supplementary material for: Induction of the pneumococcal vncRS operon by lactoferrin is essential for pneumonia
Source: Virulence. 2018 Sep 24;9(1):1562–75. doi: 10.1080/21505594.2018.1526529 (PMC6177237; doi:10.1080/21505594.2018.1526529)
Supplement: Supplemental Material [file kvir-09-01-1526529-s001.doc]

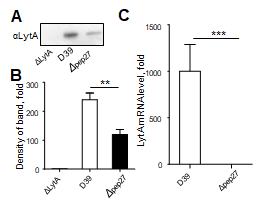


**Supplementary figure 1. Pep27 regulates LytA expression.** (A-C) 10 μg of peumococcal cell lysates from mid-log phase were used for Western blot to determine LytA level (A) Densitometric analysis of band intensity in (A) was shown (B). *lytA* mRNA was determined by qRT-PCR (C). (A-B) Representative data from 3 independent experiments were shown. (C) Experiment were repeated at least 3 times, and data are expressed as mean ± standard error of means (SEM) of experiments in triplicates. **P* < 0.05 (one-way ANOVA) as compared between groups.


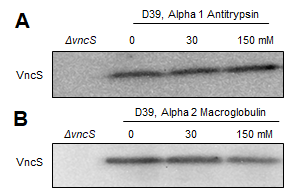


**Supplementary figure 2. Alpha 1 antitrypsin and Alpha 2 macroglobulin did not induce VncS in *S. pneumoniae***. D39 WT at mid-log phase was incubated with various concentrations of Alpha 1 antitrypsin (A) and Alpha 2 macroglobulin (B) for 10 min. Expression of VncS was determined by immunoblotting using VncR antibody.


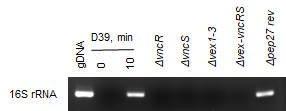


**Supplementary figure 3. Pep27 is essential for lysis**. The WT D39 and all the mutant cells in the mid-log phase were exposed to LF for 10 minutes, and the cell culture supernatant containing the released chromosomal DNA was detected by PCR using 16S rRNA primers.


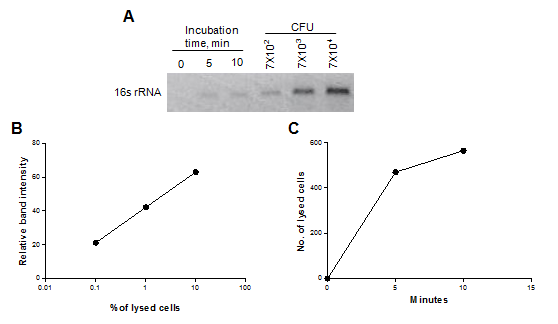


**Supplementary figure 4. Induction of lysis after LF treatment**. (A) Human serum was added to D39 culture to a final concentration 10%, and chromosomal DNA released into the culture supernatant was detected by PCR using 16s rRNA as primers (Lanes 1-3). Simultaneously, defined number of bacteria were lysed and used as a control for PCR (Lanes 4-6). A standard curve was drawn based on the relationship between the relative band density and percentage of cell lysis (B). Percentage of cell lysis as a result of serum treatment was then quantified by comparing the relative band density of PCR bands (C).
